# Comprehensive analysis and treatment of intraoral thyroglossal duct cysts with intraoperative techniques: a 7-case series review in a tertiary care center

**DOI:** 10.1016/j.bjorl.2024.101460

**Published:** 2024-06-13

**Authors:** Bo Li, Cuiping She, Delong Liu

**Affiliations:** a Department of Otorhinolaryngology Head and Neck Surgery, Dalian Municipal Central Hospital, China; b Dalian Medical University, Dalian, China

**Keywords:** Thyroglossal duct cyst, Intraoral thyroglossal duct cyst, Low-temperature plasma radiofrequency ablation, Sistrunk procedure

## Abstract

**Objective:**

Intraoral thyroglossal duct cyst is a relatively rare clinical disease. This article reviews the diagnosis and treatment process of 7 patients and explores the clinical characteristics of diagnosis and treatment of intraoral thyroglossal duct cyst in combination with past literature reports.

**Method:**

A retrospective analysis was conducted on 7 cases of intraoral thyroglossal duct cyst admitted to the Otolaryngology ward of Dalian Municipal Central Hospital from January 2017 to January 2024. The cases were recorded in terms of gender, age, symptoms, physical signs, radiological examinations, surgical methods, and postoperative complications. All cases were followed up, and the latest follow-up results were recorded.

**Results:**

Among the 7 cases, 6 patients underwent laryngoscopic and radiological examinations before surgery, and 1 child was found to have a cyst during surgery. All cases were diagnosed with intraoral thyroglossal duct cyst and treated with plasma radiofrequency surgery. None of the patients had postoperative complications, and no recurrence was found in the six-month follow-up after discharge.

**Conclusion:**

Intraoral thyroglossal duct cyst is rare in clinical practice. It is important to pay attention to its differential diagnosis clinically, and careful review of images is required before surgery. Cryoablation with low-temperature plasma radiofrequency is not only minimally invasive and has a quick recovery but also has few complications and a low recurrence rate. It is a safe and effective treatment method that is worthy of clinical promotion.

**Level of Evidence:**

Level 3.

## Highlights

• This manuscript reports on seven rare cases of intraoral thyroglossal duct cysts.
• Low-temperature plasma radiofrequency ablation surgery causes minimal trauma.
• It allows for quick recovery and has few complications and a low recurrence rate.

## Introduction

Intraoral thyroglossal duct cyst is a clinically rare disease, accounting for only 0.6%–3% of thyroglossal duct disorders.^1^, ^2^ It predominantly occurs in children but can also be seen in adults. The symptoms are complex and atypical, necessitating differentiation from various diseases. Additionally, there are diverse treatment methods. This article reviews 7 cases of intraoral thyroglossal duct cyst treated in the Otolaryngology ward of Dalian Municipal Central Hospital, combined with existing literature reports, to provide a reference for the clinical diagnosis and treatment of this condition.

## Methods

**Figure 1:**
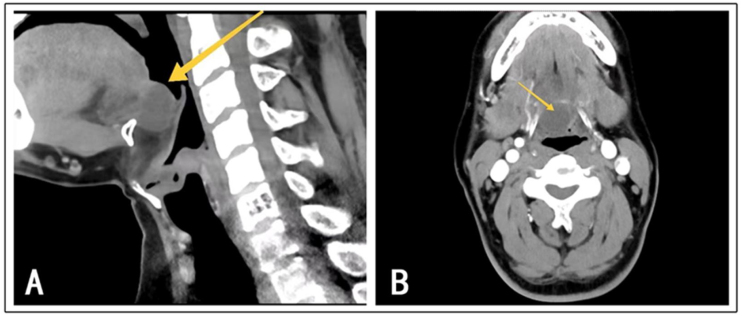
(A) Case 1: Location of the intraoral thyroglossal duct cyst as shown on the CT sagittal plane. (B) Case 1: Location of the intraoral thyroglossal duct cyst as shown on the CT axial plane.

**Figure 2:**
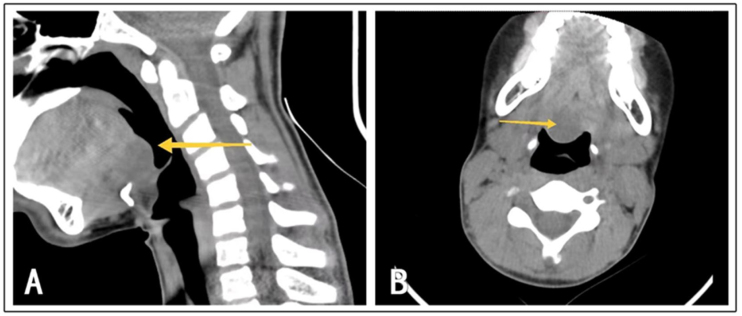
(A) Case 2: Location of the intraoral thyroglossal duct cyst as shown on the CT sagittal plane. (B) Case 2: Location of the intraoral thyroglossal duct cyst as shown on the CT axial plane.

A retrospective analysis was conducted on 7 patients with intraoral thyroglossal duct cyst treated in the Otolaryngology ward of Dalian Municipal Central Hospital from 2017 to 2023. Among these, there were 5 males and 2 females, aged between 4 and 54 years, with a median age of 11 years. The cases were documented in terms of gender, age, symptoms, physical signs, radiological examinations (Figs. 1‒2), surgical methods, and postoperative complications (Table 1). All cases were followed up, and the latest follow-up results were recorded. The Institutional Review Board (IRB) of our hospital has granted an exemption from obtaining informed consent for this retrospective case series study.

**Table 1:** Detailed information of the 7 patients in the case series.

Case nº	1	2	3	4	5	6	7
Gender	Male	Female	Female	Male	Male	Male	Male
Age	37	13	54	11	4	5	9
Symptoms	Throat discomfort accompanied by a sensation of a foreign body and a feeling of obstruction when swallowing for 1 year.	A mass was discovered during electronic laryngoscopy examination 1 week ago.	Throat pain accompanied by a sensation of a foreign body in the throat for 2 years, worsening over the past 15 days.	Throat discomfort for 1 month.	Snoring with mouth breathing during sleep, accompanied by episodes of breath-holding and waking up gasping for air for 1 month.	A throat mass was discovered during hernia surgery 6 days ago.	Snoring with mouth breathing during sleep for 1 month.
Signs	At the base of the tongue, slightly to the right of the midline near the root of the epiglottis, there is a quail egg-sized mass with a smooth surface, clear boundaries, and visible blood vessels.	A round, smooth neoplasm is visible at the base of the tongue.	There is a bulge on the left side of the base of the tongue. After clearing the secretions, a cystic cavity is observed at the bottom of the left vallecula near the base of the tongue.	A pink, smooth swelling is visible at the base of the tongue.	There is a neoplastic mass at the base of the tongue with a smooth surface, presenting in a pale-yellow color.	At the base of the tongue, slightly to the left, there is a round, smooth-surfaced neoplasm, about the size of a grape.	A smooth-surfaced mass is located in the center of the base of the tongue.
Diagnostic Method	CT	CT	CT	CT	/	CT	CT
Size of the mass	22 × 21 mm	22 × 18 mm	9 × 5 mm	10 × 10 mm	Intraoperatively, a cyst about 2 cm in diameter was observed.	5 × 5 mm	18 × 20 mm
Treatment Method	Low-Temperature Plasma Radiofrequency Ablation	Low-Temperature Plasma Radiofrequency Ablation	Low-Temperature Plasma Radiofrequency Ablation	Low-Temperature Plasma Radiofrequency Ablation	Low-Temperature Plasma Radiofrequency Ablation	Low-Temperature Plasma Radiofrequency Ablation	Low-Temperature Plasma Radiofrequency Ablation
Postoperative Complications	None	None	None	None	None	None	None
Follow-up Results	None	None	None	None	None	None	None

## Results

Table 1 summarizes the detailed information of the 7 patients with intraoral thyroglossal duct cyst. Before surgery, 6 patients underwent laryngoscopic and radiological examinations and were diagnosed with intraoral thyroglossal duct cyst. In one child, the cyst was discovered during surgery. All cases were treated with plasma radiofrequency surgery. The low-temperature plasma radiofrequency surgery system used the Arthrocare 5874# low-temperature plasma scalpel from the United States, with cutting settings at levels 7–9 and coagulation at levels 3‒5.

The patient is in the supine position. Six patients underwent nasotracheal intubation under general anesthesia, while one pediatric patient with adenoid hypertrophy underwent orotracheal intubation under general anesthesia. The patient was positioned in the supine Trendelenburg position with the head tilted downwards. The surgeon sat at the anterior side of the patient's head and used a non-visual laryngoscope to open the oral cavity and lift the tongue root, facilitating exposure of the surgical area.

**Figure 3:**
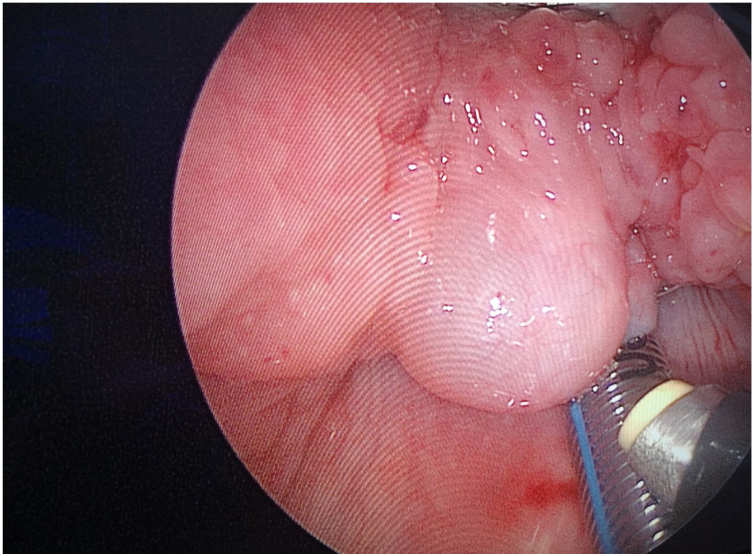
The non-visual laryngoscope is used to open the oral cavity and lift the tongue root, exposing the surgical area. A 30° nasal endoscope serves as the light source to reveal the cyst location.

Depending on the exposure of the patient's oral cavity, the shape of the plasma knife can be appropriately bent into an S-shape to better reach the surgical area and complete the procedure. A 30° nasal endoscope was used as the light source to reveal the location of the cyst (Fig. 3). The first assistant then held the anesthetic laryngoscope to maintain appropriate exposure, while the second assistant held the 30° nasal endoscope.

**Figure 4:**
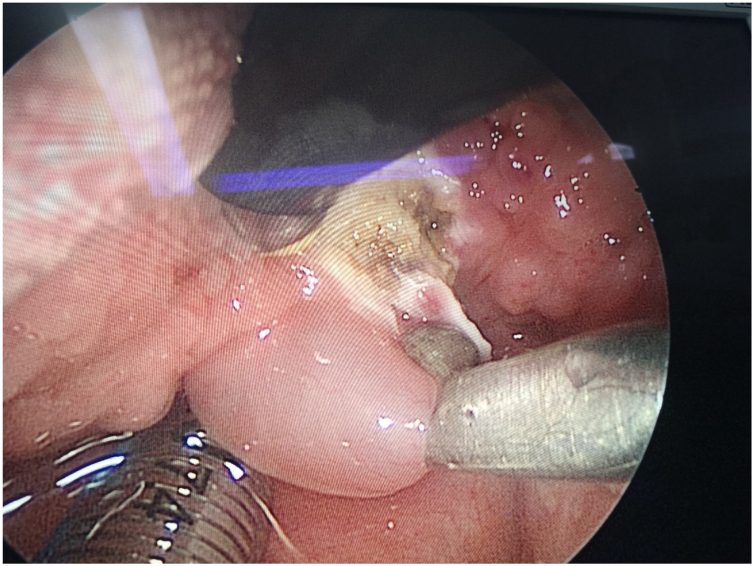
The surgeon uses laryngeal polyp forceps in the left hand to grasp the cyst wall, while the right hand employs a low-temperature plasma knife to perform a circumferential incision starting from the junction of the cyst wall and normal tissue.

**Figure 5:**
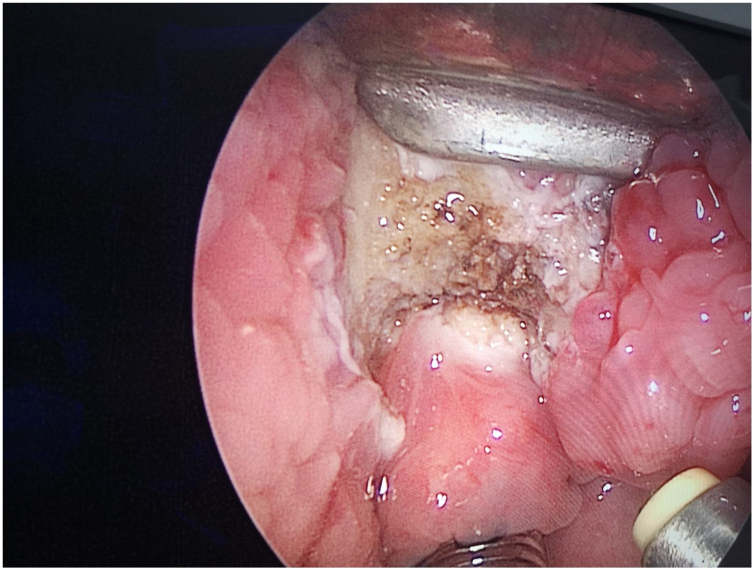
The cyst wall visible under the endoscope is completely excised until approaching the muscle layer of the tongue root. The endoscopic view after excision is shown.

The surgeon used a laryngeal polyp forceps with the left hand to grasp the cyst wall (Fig. 4) and a low-temperature plasma knife with the right hand to start circular excision from the junction of the cyst wall and normal tissue. During the excision of the cyst wall, cyst fluid would flow out, and the cyst wall visible under the endoscope was completely removed until approaching the tongue root muscle layer (Fig. 5). Minor intraoperative bleeding was directly managed with the plasma knife for hemostasis. The excised cyst wall was sent for postoperative pathological examination.

After awakening from general anesthesia, patients returned to the ward for routine nebulization and anti-inflammatory treatment. For patients with larger cysts, the cyst fluid was aspirated first to collapse the cyst and reduce tension before excision. None of the patients had postoperative complications, and no recurrence was found in the six-month follow-up after discharge.

## Discussion

Thyroglossal duct cysts are soft tissue cysts that commonly occur in children and adolescents, resulting from incomplete regression of the thyroglossal duct during embryonic development, leading to the formation of cysts due to the secretion of fluid by residual epithelial cells. The thyroid primordium initially forms in the late 4th week of gestation (around day 25) and descends to the neck along the tail end of the thyroglossal duct during development. Before birth, the thyroid moves down to its normal position below the thyroid cartilage and above the sternum in the neck. Any part of this path of thyroid movement may fail to regress completely, leaving epithelial remnants that secrete fluid and form what is known as a thyroglossal duct cyst.^3^ This can occur at any time in a person's life, even into adulthood. The incidence is higher in males than in females.^4^ In this group of intraoral thyroglossal duct cyst cases, there were 5 males and 2 females, consistent with the gender difference in the incidence of thyroglossal duct disorders.

Thyroglossal duct cysts typically occur between the hyoid bone and the thyroid gland (60.9%), above the hyoid bone (24.1%), and above the sternum (12.9%).^5^, ^6^ Occurrence within the oral cavity (at the base of the tongue) is rare, accounting for only 0.5%–3%.^1^, ^2^ The location is concealed, and when the cyst is small, symptoms are not obvious, possibly causing mild throat discomfort. However, when the cyst is large, due to its proximity to the epiglottis and glottis, it can cause a sensation of a foreign body in the throat, difficulty swallowing, snoring, and even potentially lead to breathing difficulties and asphyxiation.^6^, ^7^, ^8^, ^9^ Due to its low incidence rate, it is particularly important to prevent misdiagnosis in clinical practice. The diseases that most need to be differentiated include hypertrophy of the lingual tonsils and ectopic thyroid at the base of the tongue. Hypertrophy of the lingual tonsils is due to chronic inflammatory stimulation, leading to proliferation of lymphoid tissue at the base of the tongue, presenting as a mass (rather than a cystic lump), mainly concentrated in the vallecula and pharyngeal sidewall, causing a sensation of a foreign body in the throat and pharynx, which may disappear with swallowing and severely affect sleep breathing. The main symptoms of ectopic thyroid at the base of the tongue are a sensation of a foreign body in the throat, dry cough, difficulty swallowing, or accompanying voice disorders, and snoring at night. The physical sign is mainly a purple-red hemispherical mass visible at the base of the tongue. Preoperative ultrasound can determine the absence of the thyroid gland in the neck. In addition, this condition should also be differentiated from various benign and malignant tumors occurring in this area, including epiglottic cysts, pharyngeal fibromas, pharyngeal hemangiomas, etc. In this group of cases, Case 3 was misdiagnosed as an epiglottic cyst before surgery due to the special location of the tumor. The difference is that the cyst wall of the lingual thyroglossal duct cyst is generally pale and translucent, resembling a lychee, and fuller than an epiglottic cyst, which is generally pale yellow and more localized to the lingual surface of the epiglottis. Imaging studies for lingual thyroglossal duct cysts include ultrasound, CT, and MRI.^4^ The main purpose of ultrasound examination of lingual cysts is to differentiate them from ectopic thyroid. When reviewing CT and MRI images, physicians should focus on whether the cyst extends from the base of the tongue to the hyoid bone.^10^

The surgical approach for lingual thyroglossal duct cysts has been a subject of debate. Traditionally, the standard treatment for thyroglossal duct cysts is the Sistrunk procedure,^4^, ^11^ which includes en bloc excision of the cyst through the neck, central hyoidectomy, and conical excision of the epithelial tract and surrounding tissue from the undersurface of the hyoid bone to the foramen cecum. The recurrence rate of lingual thyroglossal duct cysts treated with the Sistrunk procedure is about 2%–6%.^12^, ^13^, ^14^ However, the Sistrunk procedure involves a wide excision range, significant surgical trauma, slow postoperative recovery, and numerous complications (including pharyngeal fistula, short-term airway obstruction, etc.), and the recurrence rate is not low.^10^ Another treatment method is the more conservative cystoplasty. Li et al.^15^, ^16^ reported some patients who underwent cystoplasty without recurrence. However, I have some doubts about cystoplasty: the bag-shaped suture partially excises the cyst, forming an open trumpet-shaped pouch towards the mouth. The residual cyst wall still has some secretory function. For adults, the secretion of cyst fluid may be expelled with swallowing or coughing without significant impact, but for infants and young children with incomplete cough and pharyngeal reflexes, could the secretion of cyst fluid affect breathing? This needs to be considered.

As mentioned above, we believe that the better treatment method for lingual thyroglossal duct cysts is surgery under oral endoscopy. This method is simple, safe, avoids neck scarring, causes minimal injury, and allows for quick patient recovery. Even if there is postoperative recurrence, it does not affect the anatomical structure of the neck and the base of the tongue, and the Sistrunk procedure can still be used.^17^ Moreover, it has fewer postoperative complications and a low recurrence rate. Nicola M. Pereira et al. conducted a systematic review of published literature on lingual thyroglossal duct cysts and found that the total recurrence rate using endoscopic or transoral techniques was 1.9%, lower than the recurrence rate of the Sistrunk procedure reported in the literature.^18^ Among them, the low-temperature plasma radiofrequency ablation technique, compared to traditional microwave, laser, and monopolar coagulation, has advantages such as protecting the mucosa, reducing surgery time, reducing intraoperative bleeding, and alleviating postoperative pain.^19^ Therefore, our department applied low-temperature plasma radiofrequency ablation technology in treating cases of intraoral thyroglossal duct cysts, achieving good therapeutic effects. All cases had no postoperative complications, and there was no recurrence in the six-month follow-up after discharge.

One of the risks of this surgery is damage to the lingual artery, especially when the cyst is deep in the tongue root muscle tissue.^18^ We believe that in such cases, the surgery should still be conservative and carefully performed. After pulling the cyst wall to see the bottom of the cyst, the plasma knife head is inserted deep into the cyst wall at the base of the tongue for electrocoagulation destruction. If the cyst is deeper and closely related to the hyoid bone, the Sistrunk procedure is recommended. Additionally, when the cyst is small, the laryngoscope placed in the mouth during surgery can easily compress and rupture the cyst. If the cyst fluid flows out, it can be difficult to locate the lingual cyst. This can be determined by moving the laryngoscope and distinguishing the color of the mucosa at the base of the tongue in combination with preoperative positioning. After the lingual cyst is ruptured and compressed by the laryngoscope, the cyst wall will appear yellow-white, different from the pale white color presented by the compressed base of the tongue.^10^

After the surgery, the supporting laryngoscope is removed, and the scope is slowly withdrawn, gradually releasing the pressure on the base of the tongue and checking for bleeding around the base of the tongue. If there is still oozing blood around the wound on the base of the tongue, careful hemostasis is needed with the plasma knife. Postoperatively, it is necessary to observe whether there is swelling at the base of the tongue and the formation of a white membrane in the surgical area. Generally, low-temperature plasma (40°‒70 °C) causes minimal thermal damage to normal tissues,^20^, ^21^ and the surgical area will not swell significantly or block the airway. Postoperatively, patients should be advised to have regular follow-ups to prevent recurrence of the tumor.

In summary, intraoral thyroglossal duct cysts are rare in clinical practice. Clinically, it is important to pay attention to differential diagnosis and to carefully review images before surgery. Low-temperature plasma radiofrequency ablation surgery not only causes minimal trauma and allows for quick recovery but also has few complications and a low recurrence rate. It is a safe and effective treatment method that is worthy of clinical promotion.

## Conclusion

Intraoral thyroglossal duct cysts are rare in clinical practice. Clinically, it is important to pay attention to differential diagnosis and to carefully review images before surgery. Low-temperature plasma radiofrequency ablation surgery not only causes minimal trauma and allows for quick recovery but also has few complications and a low recurrence rate. It is a safe and effective treatment method that is worthy of clinical promotion.

## Statements and declarations

This research did not receive any specific grant from funding agencies in the public, commercial, or not-for-profit sectors. No potential conflict of interest was reported by the authors.

## Conflicts of interest

The authors declare no conflicts of interest.
